# Involvement of the sensorimotor system in less advanced L2 processing: Evidence from a semantic category decision task

**DOI:** 10.3389/fpsyg.2022.980967

**Published:** 2022-12-01

**Authors:** Yating Bai, Wenguang He

**Affiliations:** Department of Psychology, Qufu Normal University, Qufu, China

**Keywords:** embodied cognition, L2 processing, lexical decision task, semantic category task, bilingualism

## Abstract

There is increasing evidence indicating that the sensorimotor system is involved in advanced L2 processing, which raises the question of what role sensorimotor information plays in the course of less advanced L2 comprehension. In the current study, two experiments were conducted using a lexical decision task (LDT) and semantic category task (SCT). The results showed that, in the LDT, a task more likely to result in participants making judgments based on the physical properties of words (e.g., familiarity, orthography), “up” words (e.g., *sun, plane*) did not result in faster upward than downward responses, and “down” words (e.g., *tunnel, cave*) also did not result in faster downward than upward responses. In the SCT, compatibility effects were found; specifically, searching for the up target after “up” words was faster than after “down” words and searching for the bottom target after “down” words was faster than after “upward-pointing” words. Hence, we concluded that L2 sensorimotor association, at least for L2 with low proficiency, not automatic in nature and is dependent upon deeper semantic task demands.

## Introduction

Recent research evidence has indicated that native language (L1) processing is more than a purely symbolic process (Kintsch, 1988; Glenverg and Kaschak, 2002; Felisatti et al., 2022), in which the sensorimotor system is also activated (Barsalou, 1999). For example, in studies investigating the embodied effect in sentence comprehension (Zwaan et al., 2002), researchers found that participants responded significantly faster to pictures consistent with the implied sentential content. Hence, they concluded that perceptual symbols are routinely activated in language comprehension. Similarly, a considerable number of behavioral studies found that people simulate a range of other perceptual features, such as orientation (Stanfield and Zwaan, 2001), location (Bergen et al., 2007), visibility conditions (Horton and Rapp, 2003), and motion (Kaschak et al., 2005). Neuroimaging studies also proved that language comprehension crucially involves the simulation of sensory, motor, and emotional content (De Grauwe et al., 2014; Birba et al., 2020). Recently, *f*MRI studies showed that reading action verbs reliably activated the motor cortex (Kiefer and Pulvermüller, 2011); these areas of the brain were even recruited while reading nouns expressing graspable objects (Desai et al., 2016).

Although evidence increasingly supports embodied cognition, the exact mechanism of these activations remains subject to debate. Some scholars have argued that embodied mechanisms are indeed an inseparable and functionally crucial part of language processing (Vukovic et al., 2017), but others have contended that these mechanisms might just be a by-product of language processing, functionally “redundant,” and irrelevant to efficient semantic comprehension (Kühne and Gianelli, 2019). To disentangle the above-mentioned issues, studies focusing on the embodied effects in L2 processing have been conducted. The results indicated that, as in L1 processing, the sensorimotor system is also involved in L2 processing, although some differences were found in terms of degree or time course (De Grauwe et al., 2014; Dudschig et al., 2014; Vukovic and Williams, 2014; Foroni, 2015; Buccino et al., 2017; Ratcliffe and Tokarchuk, 2020).

Based on the above findings, some researchers have argued that embodied effects are universal across L1 and L2. However, such conclusions were drawn with caution because the bilingual speakers recruited in these studies were all highly proficient. Some bilingual models, such as the Revised Hierarchical Model (RHM; Kroll and Stewart, 1994) and the Bilingual Interactive-Activation Model (BIA-d; Grainger et al., 2010), were used to show that the semantic link between the L2 and the conceptual store begins to strengthen with the development in L2 proficiency, such that eventually L1 mediation may not be necessary if a high enough level of proficiency is reached. Thus, highly proficient L2 learners have a similar representation mechanism as L1 speakers. However, for bilinguals with low proficiency, the link between L2 words and the conceptual store is weak, and L2 semantic access is accomplished *via* the activation of L1 counterparts. People with low L2 proficiency tend to be late bilinguals who acquired the L2 explicitly in a school context, in which L2 learning often takes place in a specific and limited setting, without the direct contact with events or entities described in language which takes place in L1 learning. Therefore, L2 words are claimed to have “less rich” semantic representations, i.e., they may be associated with fewer senses than L1 words or advanced L2 words. Such differences motivated us to consider whether, as with L1 processing, the sensorimotor system is also engaged in L2 processing for bilinguals with low proficiency.

Another issue still under debate is the embodied effect mechanism in L2 processing. Some studies have argued that the sensorimotor system is involved in the initial stages of L2 processing, as is the case in L1 processing. For example, using the Stroop task, Dudschig et al. (2014) found that action–sentence compatibility effects were triggered automatically for both implicit location words and emotion words in L2 processing. In a go/no-go paradigm, Buccino et al. (2017) discovered that the motor system was involved in processing nouns regarding graspable objects as compared with non-graspable ones. These studies suggested that L2 sensorimotor associations are automatic in nature and do not depend on deeper semantic task demands. However, in contrast to these accounts, studies on the simulation of language comprehension (Fischer and Zwaan, 2008; Kiefer and Pulvermüller, 2011) suggested that semantic information is kept in a distributed fashion in modality-specific sensory and motor areas. Hence, language processing is accomplished by the use of motor, perceptual, and emotional systems to simulate the situations described by the words or sentences. Thus, embodied effects in language comprehension might not reflect a process that is basic to language processing, but is rather the result of participants’ conscious decision to imagine a described scene after they have already understood the meaning. As Meteyard et al. (2012) noted, perceptual and sensorimotor information is activated when semantic representations are accessed.

In sum, there are two main perspectives on the mechanism of embodied effects in language processing. One holds that the embodied effect found in language processing is the result of the processing of the physical attributes of words (Dudschig et al., 2014), while the other contends that semantic representation plays a crucial role in the embodied effect (Fischer and Zwaan, 2008).

## Experiments

To disentangle the above-mentioned issues, a lexical decision task (LDT) and semantic category task (SCT) were implemented in the current study. In the LDT, participants were asked to decide whether the stimuli that appeared was a real word or not as quickly and accurately as possible. Due to the stimulus being presented very quickly (about 100 ms in duration), subjects did not have enough time to fully access the semantic information, and their judgments were mainly influenced by superficial attributes of words such as orthography, acoustic aspects, or word frequency. If embodied effects were found in the task, this would indicate that the effect occurs in the early stage of word processing. In the SCT, participants were asked to classify the stimuli presented. Owning to the stimuli being presented for about 800 ms, participants had enough time to access the semantics, and so according to the simulation of language comprehension, embodied effects would be found in the SCT.

In the LDT, participants are rapidly presented with some words and asked to decide whether the lexeme they saw was a true word or not; therefore, they may rely more on familiarity-based information (e.g., word frequency, orthography) to discriminate a word from a pseudo-word (Zunini and Renoult, 2017). In the SCT, participants are required to point out which category the word they saw belongs to; hence, they may have to determine the specific meaning of a word or at least require more access to semantic information than in the LDT to make a decision. Some studies have indicated that semantic information is not fully accessed in the LDT, meaning that participants’ performance is mainly affected by shallow lexical factors such as word frequency, word length, and familiarity of words (Balota and Chumbley, 1985). If we found embodied effects in the LDT, this would mean that the sensorimotor system is also involved in the processing of superficial language components such as word frequency and orthography. If the embodied effect is derived from semantic access, then a strong embodied effect would be found in the SCT but not in the LDT, because participants need to deeply access the semantic information of words in order to succeed in the SCT.

### Experiment 1

#### Methods

##### Participants

Forty-two native Chinese speakers (L1) took part in the experiment (16 male, *M*_age_ = 21.98, *SD*_age_ = 1.24); they were all late bilinguals with low proficiency in English (L2) and none of them had ever lived in an English-speaking country. The participants started learning the L2 at age 9–10. All the participants were right-handed, had normal or corrected-to-normal vision, and had no history of hearing or language difficulties or neurological/psychiatric impairment based on self-report. They signed a consent form before the experiment began and were paid for their participation. Ethical approval was given by the Committee of Protection of Subjects at Qufu Normal University. Participants were recruited based on four participant-selection criteria: duration of English language learning, College English Test-Band 6 (CET 6),^1^ Oxford placement test,^2^ and self-rating of L2 skills. The self-rating of L2 skills was based on a six-point scale assessment (ranging from 1 = “quite poor” to 6 = “highly proficient”). These tests were demonstrated to be valid measures of overall language proficiency (Hulstijn, 2012). Detailed biographical data of participants are presented in Table 1.

**Table 1:** The biographical data of the participants in low L2 proficiency group (SD).

Testing item	Rating scale (E1)	
	Experiment 1	Experiment 2
College English Test-Band 6 (CET6)	456.2 (35.8)	461.7 (36.4)
Oxford Placement Test (OPT)	40.45 (3.21)	40.65 (3.02)
Age of acquisition L2	9.38 (1.43)	9.35 (1.52)
Duration of L2 learning	11.34 (1.54)	11.36 (1.48)
Listening	2.85 (1.28)	2.98 (1.41)
Speaking	2.06 (1.32)	2.07 (1.37)
Reading	3.46 (1.35)	3.49 (1.41)
Writing	3.29 (1.57)	3.33 (1.55)

##### Materials

With reference to the existing research (Estes et al., 2007) and the purpose of the study, 60 English nouns (see Appendix) denoting different locations were used. Of them, 20 were “up” words (e.g., *roof*), 20 were “down” words (e.g., *root*), and 20 did not denote a location (e.g., *book*). Sixty pseudo-words were also selected, and were constructed by substituting one or two consonants or vowels in each noun (e.g., *griss* instead of *grass*). Through this procedure, the pseudo-words contained orthographically and phonologically permissible syllables in the English language. Words were controlled for frequency,^3^ length, and typical location (on the vertical axis). For this purpose, 20 volunteers who had passed CET 6 rated 60 true nouns on a seven-point Likert scale (1 = “very down,” 4 = “not sure about the location,” 7 = “very up”). Words selected as “down” words had rating values smaller or equal than 2.2 (*M* = 1.88, *SD* = 0.27), words selected as “up” words had rating values equal or larger than 5.6 (*M* = 6.02, *SD* = 0.4), and words that did not denote a location had rating values around 3.6–4.5 (*M* = 4.05, *SD* = 0.24). The three categories of nouns did not differ significantly with regard to frequency, *F*(2, 57) = 0.116, *p* = 0.891, or length, *F*(2, 57) = 0.42, *p* = 0.658, but did differ significantly for the rated position, *F*(2, 57) = 64.14, *p* < 0.001.

##### Procedure and design

The experiment was conducted in a sound-attenuated and dimly illuminated experiment room. Participants sat comfortably in front of a PC screen (HP 21.5′ LCD, 1,920 × 1,080-pixel resolution, and 60 Hz refresh rate) at a distance of around 60 cm. Their task was to decide whether the target word was true or not as quickly as possible.

**Figure 1:**
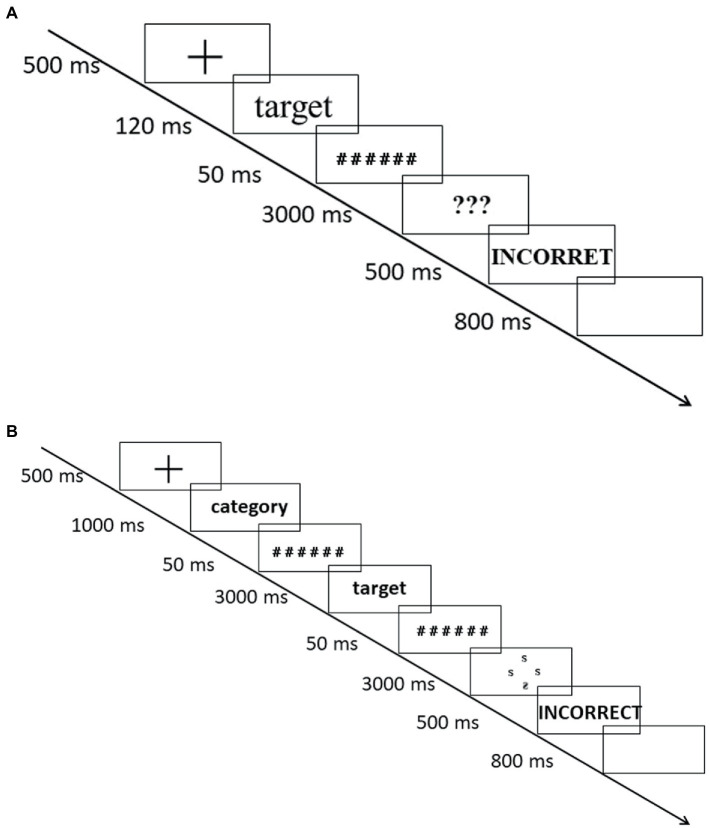
**(A)** The procedure of Experiment 1. **(B)** The procedure of Experiment 2.

Figure 1A displays the experimental procedure. Each trial began with a centrally presented fixation cross for 500 ms. Then a target word appeared at the same location for 120 ms. After the target word presentation and 50 ms for masking stimuli, three “?” appeared in the same location as the target word, cuing participants to make decisions as quickly and as accurately as possible. If the participants perceived the target word to be true, they would press the up arrow in the top part of keypad with their right index finger, or if the target word was a non-word, then they would press the down arrow in the bottom part of keypad. If the participants did not respond within 3,000 ms, the cued signal would disappear. If they were inaccurate, the word “INCORRECT” appeared in red font for 500 ms. After an inter-trial interval (800 ms) the next trial started. Eight practice trials were conducted before four blocks consisting of 120 trials each (60 trials for true words and 60 trials for non-words). Of the four blocks, two blocks required participants to press the up arrow on the keypad for true words, and the other two blocks required them to press the up arrow for non-words. Within each block, the order of presentation was randomized for each participant.

If L2-sensorimotor associations are automatic in nature and do not depend on deeper semantic task demands or L2 proficiency, cognitive advantages would be found in the congruent condition (e.g., seeing an “up” word and pressing the up arrow on the keypad) compared with the incongruent condition (e.g., seeing an “up” word and pressing the down arrow) or baseline condition (e.g., seeing a word that did not denote a location).

##### Results and discussion

The data from two participants were excluded due to the low accuracy of these participants (<80%). Erroneous trials and reaction times (*RTs*) out of 2.5 *SD* were not included in the analysis, reducing the dataset by 3.14%. The results are shown in Table 2.

**Table 2:** Reaction time (ms) and accuracy (%) in lexcial decision task (LDT).

**Response**	**Reaction time**			**Accuracy**		
	**Up-pointing word**	**Down-pointing word**	**Irrespective location word**	**Up-pointing word**	**Down-pointing word**	**Irrespective location word**
Up-response	493.76 (49.93)	491.59 (53.82)	514.94 (79.46)	90.19 (6.59)	91.31 (7.45)	90.50 (6.08)
Down-response	580.56 (84.13)	560.25 (54.53)	565.31 (73.73)	89.63 (7.31)	90.63 (7.27)	90.50 (7.25)

*RTs* were analyzed with an ANOVA with word-direction and response-direction. There was no main effect of word-direction, *F*(1, 39) = 1.64, *p* = 0.208, *η_p_*^2^ = 0.04, but the main effect of response-direction was significant, *F*(1, 39) = 10.83, *p* = 0.002, *η_p_*^2^ = 0.217, where the response to press the up arrow was faster than pressing the down arrow (492.67 ms vs. 570.71 ms). The interaction between word-direction and response-direction was not significant, *F*(1, 39) = 0.96, *p* = 0.33, *η_p_*^2^ = 0.024. A paired *t*-test between the congruent condition (e.g., seeing an “up” word and pressing the up arrow) and incongruent condition (e.g., seeing an “up” word and pressing the down arrow) also indicated no significance, *t*(81) = 1.08, *p* = 0.285, Cohen’ s *d* = 0.055. Analysis of accuracy showed that no significant differences were found, *FS < 1*.

In sum, no differences were noted in Experiment 1, suggesting that L2 word decisions could not activate spatially directed motor response automatically for late L2 learners with low proficiency. The results are inconsistent with findings from Dudschig et al. (2014). Using the vertical Stroop paradigm and similar materials, they found that L2 processing automatically activated motor responses similar to L1 processing for late L2 learners; participants were not required to actively read or evaluate word meaning. They concluded that L2 sensorimotor associations are automatic in nature and do not depend on deeper semantic task demands. Although participants in Dudschig et al.’s (2014) study were late L2 learners, they may have been highly proficient in the L2 (especially at the level of written word identification), because English shares more similarities with German, compared with Chinese and English. Current cumulative evidence arguing in favor of the involvement of the sensorimotor system in L2 processing have mainly involved semantic tasks. Thus, a further investigation was conducted in Experiment 2 using a semantic category decision task. If the L2 sensorimotor system is the result of accessing semantic representations, then cognitive advantages would be found in congruent conditions compared with incongruent conditions or baseline conditions.

### Experiment 2

#### Methods

##### Participants

Forty native Chinese speakers (L1) took part in the experiment (12 male, *M*_age_ = 21.80, *SD*_age_ = 1.32); they were all late bilinguals with low proficiency in English (L2) and none of them had ever lived in an English-speaking country or taken part in Experiment 1. Participants had started learning the L2 at age 9–10. All the participants were right-handed, had normal or corrected-to-normal vision, and had no history of hearing or language difficulties or neurological/psychiatric impairment based on self-report. The other procedure was the same as in Experiment 1. Detailed biographical data of the participants are presented in Table 1.

##### Materials

Sixty true nouns, as used in Experiment 1, were mainly from six categories: nature entities (e.g., *sun*/*cloud*/*river*), living entities or organisms (e.g., *bird*/*leg*/*seed*/*grass*/*root*), household items (e.g., *hat*/*glass*/*cake*/*wallet*), buildings (e.g., *roof*/*ceiling*/*tomb*/*tunnel*/*well*/*cave*), food (e.g., *bread*/*soap*/*cookie*), and aircraft or vehicle (e.g., *plane*/*kite*/*wheel*).

##### Procedure and design

Figure 1B displays the experimental procedure. Each trial began with a centrally presented fixation cross for 500 ms, and then a cue of the category (e.g., natural entities) appeared at the same location for 1,000 ms. After cue presentation and 50 ms for the pre-masked stimuli, the signal target (e.g., *sun*) appeared at the same location for 800 ms. After 50 ms of post-masked stimuli, three normal “S” and an inverted “S” arranged into a cross (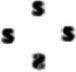) appeared in the center of the frame. If participants considered the target word to be from the category presented, they would search for the inversed “S” by pressing the corresponding arrow on the keypad. If the inversed “S” was at the bottom of the screen, participants would press the down arrow, and they would press the up arrow if the inversed “S” was at the top of the screen. If the target word was not from the category presented, participants did not need to respond and the stimulus would disappear in 3,000 ms. Eight practice trials were performed before four blocks consisting of 120 trials each. The location of the inverted “S,” participants’ response, and the matching of the target word and its category were well counterbalanced. Within each block, the order of presentation was randomized for each participant.

##### Results and discussion

Due to exploring only the embodied effects in upward or downward location word processing in our study, the data of left or right trials were not collected. Erroneous trials and reaction times (*RT*s) for “up” or “down” trials out of 2.5 *SD* were excluded from the analysis, reducing the data set by 2.42%. The results are shown in Table 3.

**Table 3:** Reaction time (ms) and accuracy (%) in semantic category decision (SCT).

**Response**	**Reaction time**			**Accuracy**		
	**Up-pointing word**	**Down-pointing word**	**Irrespective location word**	**Up-pointing word**	**Down-pointing word**	**Irrespective location word**
Up-response	891.39 (94.05)	1109.88 (78.18)	987.56 (42.89)	97.46 (2.12)	97.75 (2.49)	96.31 (4.47)
Down-response	955.37 (51.47)	1027.64 (84.13)	970.20 (75.44)	96.75 (3.17)	96.68 (3.53)	97.00 (2.33)

*RT*s were analyzed with an ANOVA with word-direction and response-direction. The main effect of word-direction was significant, *F*(2, 78) = 17.86, *p* < 0.001, *η_p_*^2^ = 0.31. Planned comparisons showed that there was a significant difference between the congruent (upward-location words and pressing the up arrow or downward-location words and pressing the down arrow) and the incongruent condition, *t*(117) = −4.83, *p* < 0.001, and the difference between the incongruent condition and baseline condition (words not denoting a location and pressing the up or down arrow) was also significant, *t*(117) = 2.98, *p* < 0.01, but the difference between the congruent and baseline condition was marginal, *t*(117) = 1.85, *p* = 0.07. The main effect of response-direction was not significant, *F* < 1. The interaction between word-direction and response-direction was significant, *F*(2, 78) = 3.56, *p* < 0.05, *η_p_*^2^ = 0.08. Simple effects analyses indicated that searching for the inversed “S” at the top of the screen following “up” words was faster than searching for the inversed “S” at the top of the screen following “down” words, *F*(1, 39) = 3.31, *p* < 0.05,*η_p_*^2^ = 0.07, and searching for the inversed “S” at the bottom of the screen following “down” words was faster than that following “up” words, *F*(1, 39) = 11.18, *p* < 0.01, *η_p_*^2^ = 0.27. Analyses of the accuracy suggested that there were no significant differences for the main and interaction effects, *FS* < 1.

The results of Experiment 2 strongly suggested that location information was indeed activated in the SCT, since responses were faster when the word’s referent location in the world was compatible with the participant’s response movement. This replicates previous findings regarding the effects of implicit location words and indicates that spatial experiential traces are activated in various tasks involving semantic retrieving (Parker Jones et al., 2012; Vukovic and Shtyrov, 2014; Vukovic and Williams, 2014; Buccino et al., 2017). Using the semantic judgment task, Qian (2016) investigated the embodied effects in processing nouns with high or low power, and the results showed that responses were faster for power words presented in the upper (vs. lower) part of the screen even for L2 speakers with low proficiency.

## General discussion

Studies have increasingly suggested that L2 processing relies on embodied representations of meaning and is connected to motor and perceptual processing, as is found in L1 processing (De Grauwe et al., 2014; Dudschig et al., 2014; Buccino et al., 2017; Gianelli et al., 2018). However, there remained some unresolved issues, for example, whether the sensorimotor system is automatically involved in L2 processing or not. The question remained as to the role of language proficiency in embodiment effects. In the current study, two experiments were conducted using the LDT and SCT. The results showed that, in the LDT, a task where participants are more likely to make judgments based on physical properties of words (e.g., familiarity, orthography), “up” words did not result in faster upward than downward responses, and “down” words also did not result in faster downward than upward responses. In the SCT, compatibility effects were found; specifically, searching for the target located at the top of the screen after “up” words was faster than after “down” words and searching for the target at the bottom of the screen after “down” words was faster than after “up” words. Hence, we concluded that L2-sensorimotor association, at least for L2 speakers with low proficiency, was not automatic in nature and did depend on deeper semantic task demands (Qian, 2016).

Increasing evidence has suggested that L2 processing is also based upon modal experiences, and is not separate from the sensory system (Dudschig et al., 2014; Buccino et al., 2017); however, it remained open whether the sensorimotor system is involved in L2 processing or not (Monaco et al., 2019). Some studies argued that L2 processing is “disembodied,” and considerable differences between L1 and L2 were noted in the literature such as age of acquisition (AOA), style of learning, and proficiency. Using the LDT, motor and non-motor cognate or non-cognate verbs in Dutch were presented to participants with highly proficient L1-German L2-Dutch and Dutch native speakers. The results indicated a significantly stronger activation in motor and somatosensory areas for motor verbs, regardless of the cognate status of the verbs. This was true of both language groups. De Grauwe et al. (2014) consequently suggested L2 representations to be rich enough to activate similar motor-related areas to L1. However, in contrast to their findings, we did not find an embodiment effect in our experiment using the LDT. One of the reasons for the difference between the two studies may relate to the participants. In our experiment, all the participants were of low proficiency and were late L2 learners. Unlike advanced L2 learners who have similar semantic representations to L1 speakers, L2 learners with low proficiency have a coarse semantic representation, in which the concept representation is detached from the sensorimotor system and environments; thus, some scholars have argued that L2 processing, especially for L2 learners with low proficiency, is “disembodied” (Pavlenko and Aneta, 2017). Another reason may be the research method. Compared with the *f*MRI method used in De Grauwe et al.’s study, the behavior method was not sensitive enough to detect the involvement of the sensorimotor system in L2 processing. A third reason may come from the similarity between the L1 and L2. In the *f*MRI study, German and Dutch are highly related languages with a large number of cognates, i.e., words with similar form and meaning in the two languages; thus, there was a high overlap between L1 and L2 representation. In our study, Chinese and English are from different language families, meaning that few common words were shared between them.

In the SCT, a stronger embodiment effect was discovered, which was consistent with many other findings. Using the picture-word mapping paradigm, Bergen et al. (2010) found both groups (L1 and L2) responded more rapidly when the picture and the word were matched. In another study (Xue et al., 2015) using EEG techniques, written high and low BOI (body-object interaction) words embedded in segmented sentences characterized by rich and poor sensorimotor context were presented, and participants were asked to make judgments about the acceptability of the sentences. The results showed that action-and perception-related brain areas for L2 words were activated, which indicated that the semantic representations for L2 are plentiful enough for sensorimotor-related activation. Combining the results of the two experiments and other studies, we concluded that the sensorimotor system is also involved in L2 processing, even for L2 learners with low proficiency.

Another key issue subject to serious debate in L2 processing is whether the sensorimotor system is involved automatically in L2 processing or is the result of consciously imagining a described scene after accessing lexical-semantic information. Some studies, such as Dudschig et al. (2014) and Buccino et al. (2017), held that the sensorimotor system is involved automatically in L2 processing and does not depend on deeper semantic task demands. Our results are not consistent with their findings because no embodiment effect was found in the LDT, in which participants made decisions according to the familiarity or form of the word, without needing to access semantic information. The sensorimotor system is automatically involved in L1 processing because, as is widely known, L1 is learned interactively and we often perceive the events and entities or feelings described. Thus, language percepts are typically combined with specific gestures, eye movements, and physical orientation toward the described entity. When seeing a target word, the sensorimotor system, events, and feelings associated with the word would be automatically activated. In contrast, L2 learning in school typically takes place in a very limited setting, whereby interactions with other people and physical experiences are less dominant during the object of inquiry, which is in large part an internal, mental process. In such a view of L2 learning, there is a division between the mind and world, especially for L2 speakers with low proficiency; thus, it is difficult to automatically activate the sensorimotor system or the referents in L2 processing. In contrast, in the SCT, a task which entailed deeper semantic processing, a strong embodiment effect was found. Taken together, we contend that the embodiment effect in L2 processing is not an automatic consequence, but the result of participants consciously imagining a described scene after they have already understood the meaning.

The following question emerges: Why is the sensorimotor system or experiential trace activated automatically in L2 processing for advanced L2 learners, but not for L2 learners with low proficiency? According to RHM (Kroll and Stewart, 1994), when L2 is still emerging, L1 mediates L2 access to the conceptual store. If this is the case, then a large amount of time would be needed to activate the sensorimotor experiential associations, because the equivalent L1 lexeme would be first retrieved, followed by access to the concept *via* the L1, and this would entail later sensorimotor involvement compared with advanced L2 learners. With the development of L2 proficiency, a semantic link begins to strengthen between the L2 and the “conceptual store,” such that, eventually, L1 mediation may not be necessary if a high enough level of proficiency is achieved and it is possible to activate the sensorimotor experiential associations instantly by accessing the conceptual store. Emerging research has demonstrated that embodiment processes occur similarly to L1 for highly proficient bilinguals, but may differ in some ways for less proficient L2 learners.

In our opinion, our findings have significant implications for debates both in embodied cognition and bilingual processing. First, although there is increasing evidence indicating that L2 comprehension is achieved by recruiting the very same resources which are used for action, perception, and emotion, most of these studies focused on advanced L2 learners, meaning it was difficult to conclude that the embodiment effect was universal in L2 processing. Our findings gave support to the statements mentioned above. Second, the embodied experience and sensorimotor system should be taken into account while constructing models of L2 representation. Third, if sensorimotor experience, emotion, and settings of lexical learning are essential to L2 processing, teachers should adopt experience-based teaching methods and encourage learners to use bodies, actions, imaginaries, and settings in L2 learning. Of course, there are still some limitations to our study. First, two different tasks were used in Experiment 1 and Experiment 2, which made it difficult to compare the results between the two experiments. Second, the absence of an embodiment effect in Experiment 1 may be due to an insufficiently sensitive approach. In future research, we will use a more sensitive approach to examine the embodied effects in L2 processing, such as by using ERPs and *f*MRI.

In conclusion, the present findings have demonstrated that the sensorimotor system is also involved in less advanced L2 processing, but this outcome was only found in the SCT and not the LDT. This suggests that the sensorimotor system is not automatically involved in L2 processing, but is the result of consciously simulating the referents after accessing semantic information.

## Data availability statement

The original contributions presented in the study are included in the article/supplementary material, further inquiries can be directed to the corresponding author.

## Ethics statement

The studies involving human participants were reviewed and approved by the Center of Experiment, Qufu Normal University. The patients/participants provided their written informed consent to participate in this study.

## Author contributions

YB performed the experiment, wrote the manuscript, and analyzed the data. WH proposed the research idea and experimental design. All authors contributed to the article and approved the submitted version.

## Funding

This research was funded by the Natural Science Fund of Shandong Province to WH (grant number: ZR2019MC048) and Higher Education Youth Innovation Science and Technology Support Program of Shandong Province (2019RWF005).

## Conflict of interest

The authors declare that the research was conducted in the absence of any commercial or financial relationships that could be construed as a potential conflict of interest.

## Publisher’s note

All claims expressed in this article are solely those of the authors and do not necessarily represent those of their affiliated organizations, or those of the publisher, the editors and the reviewers. Any product that may be evaluated in this article, or claim that may be made by its manufacturer, is not guaranteed or endorsed by the publisher.

## Supplementary material

The Supplementary material for this article can be found online at: https://www.frontiersin.org/articles/10.3389/fpsyg.2022.980967/full#supplementary-material

## Footnotes

^1^The CET 6, designed by the Ministry of Education of China, is used in all universities in China to evaluate the English proficiency of non-English majors. It consists of tasks on listening comprehension, reading comprehension, vocabulary knowledge, grammar knowledge, and writing. The total score is 710, and the cutoff point (set by the Ministry of Education) for success and failure in the test is 427.

^2^The Oxford Placement Test (OPT) includes 25 multiple-choice questions and a cloze test, and the total score is 50.

^3^ http://subtlexus.lexique.org
